# Detecting and classifying neurotransmitter signals from ultra-high sensitivity PET data: the future of molecular brain imaging

**DOI:** 10.1088/1361-6560/ac195d

**Published:** 2021-08-24

**Authors:** Heather Liu, Evan D Morris

**Affiliations:** 1 Dept. Biomedical Engineering, Yale University, New Haven, CT, United States of America; 2 Dept. Radiology and Biomedical Imaging, Yale University School of Medicine, New Haven, CT, United States of America; 3 Dept. Psychiatry, Yale University School of Medicine, New Haven, CT, United States of America

**Keywords:** next-generation, brain PET, kinetic modeling, lp-ntPET, dopamine, signal classification

## Abstract

Efforts to build the next generation of brain PET scanners are underway. It is expected that a new scanner (NS) will offer an *order-of-magnitude improvement* in sensitivity to counts compared to the current state-of-the-art, Siemens HRRT. Our goal was to explore the use of the anticipated increased sensitivity in combination with the linear-parametric neurotransmitter PET (lp-ntPET) model to improve detection and classification of transient dopamine (DA) signals. We simulated striatal [^11^C]raclopride PET data to be acquired on a future NS which will offer ten times the sensitivity of the HRRT. The simulated PET curves included the effects of DA signals that varied in start-times, peak-times, and amplitudes. We assessed the detection sensitivity of lp-ntPET to various shapes of DA signal. We evaluated classification thresholds for their ability to separate ‘early’- versus ‘late’-peaking, and ‘low’- versus ‘high’-amplitude events in a 4D phantom. To further refine the characterization of DA signals, we developed a weighted k-nearest neighbors (wkNN) algorithm to incorporate information from the neighborhood around each voxel to reclassify it, with a level of certainty. Our findings indicate that the NS would expand the range of detectable neurotransmitter events to 72%, compared to the HRRT (31%). Application of wkNN augmented the detection sensitivity to DA signals in simulated NS data to 92%. This work demonstrates that the ultra-high sensitivity expected from a new generation of brain PET scanner, combined with a novel classification algorithm, will make it possible to accurately detect and classify short-lived DA signals in the brain based on their amplitude and timing.

## 1. Introduction

The current generation of high-performance brain PET scanners is now over 20 years old (Schmand *et al* 1999). Thanks to innovations in solid-state detector technology, a new generation of advanced brain scanners is now possible. There are a number of efforts presently underway to design and build the next generation of scanners which, it is hoped, will achieve considerable advances in detection sensitivity and/or improved spatial resolution. Through the BRAIN Initiative grant mechanism, the National Institutes of Health (NIH) has recently funded three separate efforts to design and produce new brain PET devices (Catana 2018, El Fakhri 2018, Carson and Qi 2020). One is to be a PET insert into a MR/PET system, and so has its own unique set of design constraints (Catana 2018). The other two are to be free-standing PET scanners. Of these, one (Carson *et al* 2021) prioritizes sensitivity gains over resolution gains whereas the other (El Fakhri 2018) does the opposite. In general, there is a trade-off between maximal sensitivity and maximal spatial resolution (Vandenberghe *et al* 2020). High sensitivity is maximized, in great part, by extending the axial extent of the scanner as has been accomplished with great success at the whole-body level by the recently introduced uEXPLORER system (Spencer *et al* 2020). An extended axial field of view (FOV) succeeds by increasing the solid angle and capturing more axially oblique lines of response (LORs). Extended axial FOV scanners also harness time of flight information to further improve effective sensitivity and depth of interaction (DOI) to maintain good axial resolution (Zhang *et al* 2018). But these additional techniques impose a practical lower limit on the size and aspect ratio of the crystals. In contrast, a scanner designed to prioritize high resolution in a limited FOV can be designed with smaller crystals, rely primarily on direct LORs, and limited axial extent, but must ultimately sacrifice some sensitivity in doing so.

For many years, the focus of our research group has been on extracting temporal variation in neurotransmitter level from PET time activity curves (TACs) through the use of image pre-processing and advanced kinetic model fitting. A limitation of current technology has been the low signal to noise ratio (SNR) of voxel-level TACs. The promise of increased sensitivity to radioactive events, and hence better SNR, represents a potential boon for neurotransmitter studies with PET. One of the new brain scanner projects (Carson and Qi 2020) expects to achieve an order of magnitude improvement in sensitivity over the current state of the art, Siemens High Resolution Research Tomograph (HRRT). Because the next generation scanners are still in the design phase, we chose to ask a broad question: How could we leverage a ten-fold improvement in scanner sensitivity over the HRRT, without any further assumptions of improvements in performance? The present work focuses solely on the potential impact of a (generic) ultra-high sensitivity brain PET scanner (referred to here as the ‘NS’ for New Scanner), on neurotransmitter imaging.

Knowledge of voxel-level DA dynamics could offer novel insights into the transmission of information within the limbic system. It has been hypothesized that the timing and magnitude of DA release in the limbic system encode specific functions and behavioral outcomes related to reward, addiction, and cognitive control (Rodriguez de Fonseca and Navarro 1998, Adinoff 2004, Rajmohan and Mohandas 2007). Preclinical cyclic voltammetry experiments have produced evidence that a variety of kinetic domains exist simultaneously within the dorsal striatum (Taylor *et al* 2015, Walters *et al* 2016); each domain’s DA signal has a unique amplitude and temporal signature. The dynamics of the DA signal may be altered by drug interactions. Microdialysis experiments in rodents and non-human primates have shown that the amplitude of DA release exhibits a dose-dependent response to drug stimuli (Narendran *et al* 2014). Further, the temporal characteristics of DA release can be modulated with various forms of treatment before the stimuli, such as priming with another drug (Rollema *et al* 2007) or *ad libitum* self-administration of the drug for an extended period (Kirkland Henry *et al* 2009). DA dysfunction leads to psychiatric diseases such as addiction, attention-deficit hyperactivity disorder, depression, and schizophrenia (Dichter *et al* 2012). However, the spatiotemporal neurochemical signals underlying these emergent dysfunctional behaviors have not been adequately explored in clinical populations—largely due to the invasive nature of cyclic voltammetry and microdialysis. We anticipate that voxel-level lp-ntPET analysis of ultra-high sensitivity PET data could be used as a noninvasive method for detecting and characterizing different DA signals.

We sought to understand whether the expected high sensitivity of the NS could be used to further the study of neurotransmitter (NT) dynamics at voxel-level resolution. The effect of competition, between time-varying NT concentration and a radioactive tracer, on the PET signal has been studied, theoretically and experimentally (Morris *et al* 2005, Constantinescu *et al* 2008, Morris *et al* 2008, Normandin and Morris 2006, 2008, Normandin *et al* 2012, Morris *et al* 2013, Kim *et al* 2014, Wang *et al* 2017). A suite of neurotransmitter PET (‘ntPET’) models created by our group can be used to estimate parameters that describe transient stimulus-induced NT release. Linear parametric ntPET (lp-ntPET) is the linearized version of the ntPET model. Thanks to linearization, lp-ntPET can be implemented to perform voxel-level parameter estimation with high computational efficiency. lp-ntPET can been used both to detect and to characterize transient NT signals. The model has been useful in identifying dopamine (DA) voxels within the striatum that activate in response to cigarette smoking (Cosgrove *et al* 2014). Recently, we used the voxel-by-voxel approach to identify differences in the spatial pattern of dopamine response to cigarette smoking under a nicotine patch compared to the placebo condition (Zakiniaeiz and Liu *et al*, 2021submitted). However, investigators have had limited success using lp-ntPET to consistently estimate timing dynamics of the activated voxels, due to low SNR in voxel-level data from the HRRT (Hu *et al* 2019, Bevington *et al* 2020, Fuller *et al* 2020).

In this work, we used simulations to investigate whether the future NS scanner could be used with the lp-ntPET model to recover timing and amplitude information from detected DA signals. We evaluated the detection sensitivity of lp-ntPET to each DA signal (*DS*_*DA*_) within simulated NS PET data, over a range of plausible DA signals. We also assessed the ability of the model to characterize the signals based on their amplitude and peak-time. Given the anticipated ultra-high detection sensitivity to radioactive counts (*DS*_*counts*_) of the scanner, we also explored whether information from the neighborhood immediately surrounding a voxel could be used to improve detection and characterization of DA signals. For the purposes of this study, we have taken it as a given that future scanners will achieve a 10-fold improvement in *DS*_*counts*_ in every voxel of the brain, and that this increase can be translated into a 10-fold decrease in variance. We hypothesize that the 10-fold gain in *DS*_*counts*_ would provide a considerable increase in *DS*_*DA*_. The improved *DS*_*DA*_ should allow for the characterization of voxel-level DA signals as low- or high-amplitude, and as early- or late-peaking.

## 2. Methods

### 2.1. Simulations of high-count PET data from a future NS scanner

Simulations of [^11^C]raclopride (RAC) PET data in the striatum were performed in the presence of various endogenous DA signals. Radiotracer delivery was simulated as a bolus injection. Parameters and simulation methods used to produce 90 min RAC TACs with the full ntPET model (Fisher *et al* 1995, Morris *et al* 1995) are as described in Liu *et al* (Liu and Morris 2020). All simulations were implemented in MATLAB software (R2017a, The MathWorks, Inc., Natick, MA) using COMKAT modeling routines (Muzic and Cornelius 2001). DA signals were modeled with a gamma variate function according to the formulation of Madsen *et al* (1992). The gamma variate used is shown in equation (1)

\begin{eqnarray*}{h}\left({t}\right)={\left(\displaystyle \frac{{t}-{{t}}_{{d}}}{{{t}}_{{p}}-{{t}}_{{d}}}\right)}^{\alpha }\exp \left(\alpha \left(1-\,\displaystyle \frac{{t}-{{t}}_{{d}}}{{{t}}_{{p}}-{{t}}_{{d}}}\right)\right){u}\left({t}-{{t}}_{{d}}\right).\end{eqnarray*}

*h*(*t*) is the normalized DA signal over time and is in the range [0, 1]. Each DA signal is parametrized by *t*_*d*_, the signal start-time relative to injection, *t*_*p*_, the peak-time relative to injection, and $\alpha ,$ the baseline recovery rate; *u*(*t*) is the unit step function. For the Pilot Studies, DA signals were simulated with the following parametric ranges: *t*_*d*_ = [35 min, 44 min] in 3 min intervals, *t*_*p*_ = [40 min, 65 min] in 5 min intervals, and *α* = 0.5. *α* = 0.5 was chosen based on the temporal profile of DA release determined from microdialysis experiments (Carboni *et al* 1989, Laruelle *et al* 1997, Morris *et al* 2008, Jedema *et al* 2014). Total DA release, DA(t), is described by

\begin{eqnarray*}{\mathrm{DA}}\left({t}\right)={G}\,* \,{h}\left({t}\right)+{\mathrm{baseline}},\end{eqnarray*}

where, *G* is the concentration of DA at its peak above baseline concentration, and *h*(*t*) is the normalized DA signal as defined in equation (1). All signals were simulated with *G* = [50 nM, 600 nM] in 50 nM intervals. Baseline DA concentration was set to 100 nM. Hereafter, we will refer to *G* in terms of its percentage above baseline (*G*_%_), for better intuitive feel.

For each combination of DA signal parameters, 1000 noisy PET TACs were generated for the HRRT and NS scanner (using the assumption that spatial resolution is comparable for both scanners). Noise was applied independently to each TAC using methods described in Liu and Morris (2020). For each time point in the TACs, additive noise was selected from a normal distribution with zero-mean and variance proportional to the mean radioactivity signal as first introduced by Mazoyer *et al* and subsequently used widely by others (Mazoyer *et al* 1986, Liu and Morris 2020). Noise applied to the simulated HRRT data was scaled to coincide with voxel-level measurement noise observed in human RAC data acquired on the HRRT. Figure S6 in the supplemental document compares a simulated voxel TAC and a human voxel TAC taken from the striatum. Noise applied to the simulated NS scanner data was scaled such that $Var(PE{T}_{NS})=0.1* Var(PE{T}_{HRRT}).$ Measurement noise was not added to the reference region (cerebellum); exact knowledge of the cerebellum TAC was assumed. Data were binned into 3 min frames.

### 2.2. Implementation of the lp-ntPET model for estimation

The operational equation for lp-ntPET is shown in equation (3).

\begin{eqnarray*}{C}_{T}\left(t\right)=\,{R}_{1}{C}_{R}\left(t\right)+\,{k}_{2}\displaystyle {\int }_{0}^{t}{C}_{R}\left(u\right)du-{k}_{2a}\displaystyle {\int }_{0}^{t}{C}_{T}\left(u\right)du-\gamma \displaystyle {\int }_{0}^{t}{C}_{T}\left(u\right){h}_{i}\left(u\right)du.\end{eqnarray*}

*C*_T_ and *C*_R_ are the concentrations of tracer in the target and reference tissues, respectively. The target tissue contains the effect of the NT signal, and the reference tissue is used as a proxy for the input function of the tracer. *R*_1_ is the ratio of tracer delivery to the target and reference tissues; *k*_2_ is the first-order efflux rate constant from the reference tissue*; k*_2a_ is the first-order effective efflux rate constant from the target tissue, and incorporates the effect of specific binding of the tracer to the target tissue. *γ* is the magnitude of the NT component. Note that the parameters describing the magnitude of the DA signal in the simulation and estimation models are defined differently. In the simulation model, *G* is defined explicitly as a concentration of NT. In the estimation model, as a consequence of model simplification, *γ* is defined in the same units as *k*_2*a*_. ${{h}}_{{i}}\left({u}\right)$ is the normalized shape of the NT signal, selected from a library of basis functions. Each basis function takes the form described in equation (1). *t*_*d*_, *t*_*p*_, and $\alpha $ are determined by selection of the optimal basis function, as described in Normandin *et al* (2012).

Estimated NT parameters are retained at a voxel only if inclusion of ${{h}}_{{i}}\left({u}\right)$ significantly improves the fit, as determined by a model comparison metric. Without the last term on the right-hand-side, equation (3) reduces to the time-invariant model, MRTM (Ichise *et al* 2003). Equation (4) defines the Bayesian information criterion (BIC), the model comparison metric used in this work

\begin{eqnarray*}BI{C}_{M}={p}_{M}\,\ast \,\mathrm{ln}\left(n\right)+n\,\ast \,\mathrm{ln}\left(\displaystyle \frac{SS{E}_{M}}{n}\right)\,\,\,.\end{eqnarray*}

*p* is the number of parameters in model *M*; *n* is the number of data points; *SSE*_*M*_ is the sum of squared errors from the fit. $BI{C}_{MRTM}$ is calculated with ${p}_{MRTM}$ = 3. $BI{C}_{lpntPET}$ is determined with the *effective* number of parameters, ${p}_{lpntPET}^{eff},$ which has been shown to be in the range [4, 7]. ${p}_{lpntPET}^{eff}$ depends on the number of basis functions as well as the desired significance level (Liu and Morris 2020). For each pair of fits by lp-ntPET and MRTM to a single data set, we define:

\begin{eqnarray*}{\mathrm{\Delta }}BIC=BI{C}_{lpntPET}^{eff}-BI{C}_{MRTM},\end{eqnarray*}

where, $BI{C}_{lpntPET}^{eff}$ is the BIC value for the fit by lp-ntPET and $BI{C}_{MRTM}$ is the BIC value for the fit by MRTM. An estimated NT response is deemed significant only if ${\mathrm{\Delta }}BIC$ is less than zero.

All TACs were fitted with both MRTM and lp-ntPET. lp-ntPET was implemented with 38 bases (*t*_*d*_ = [35 min, 45 min], *t*_*p*_ = [35 min, 70 min], *α* = 0.5). The basis function library can be visualized in figure S1 (available online at stacks.iop.org/PMB/66/175007/mmedia) in the supplemental document.

### 2.3. Pilot study 1: *DS*_*DA*_ of a future NS scanner to DA events

To predict the *DS*_*DA*_ to DA events of a range of shapes and amplitudes, *DS*_*DA*_ was determined for all signals simulated with *t*_*d*_ = 35 min, which was previously determined to be the optimal time for detecting a brief drug stimulus in RAC data with lp-ntPET (Wang *et al* 2017). Signals peaked 5–30 min after start of stimulus (*t*_*p*_ = [40 min, 65 min]) with *G*_%_ = [50%, 600%] above baseline. *DS*_*DA*_ was calculated according to equation (6)

\begin{eqnarray*}D{S}_{DA}=\,\displaystyle \frac{\displaystyle {\sum }_{i=1}^{k}\left[{\mathrm{\Delta }}BI{C}_{i}< 0\right]}{k}\,\times 100 \% ,\end{eqnarray*}

where, *k* is the total number of data sets simulated with the same DA parameters but independent noise. Here, *DS*_*DA*_ is the percentage of significant DA responses detected out of 1000 TACs, for each combination of *t*_*p*_ and *G*_%_.

A range of signals that were identified as marginally detectable in the HRRT, but reliably detectable in the NS were identified as candidate signals for inclusion in phantom simulations.

### 2.4. Pilot study 2: performance of classification thresholds for peak-time and amplitude

Thresholds for estimated peak-time and amplitude were assessed for their ability to separate ‘early’- and ‘late’-peaking signals, and ‘low’- and ‘high’-amplitude signals, over a physiologically plausible range for a moderate drug stimulus. DA signals were simulated with the following parametric ranges: *t*_*d*_ = [35 min, 45 min], *t*_*p*_ = [40 min, 65 min], and *G*_%_ = [200%, 400%] above baseline. lp-ntPET was used to estimate DA parameters from each TAC. The timing of estimated responses was classified according to:

\begin{eqnarray*}Early:\widehat{{t}_{p}}< {t}_{p}^{* }\end{eqnarray*}

\begin{eqnarray*}Late:\widehat{{t}_{p}}> {t}_{p}^{* },\end{eqnarray*}

where $\widehat{{t}_{p}}$ is the estimated value for ${t}_{p}$ and ${t}_{p}^{* }$ is the critical peak-time separating early and late events. The amplitude of estimated responses was classified according to:

\begin{eqnarray*}Low:\displaystyle \frac{\hat{\gamma }}{\widehat{{k}_{2a}}}< M* \end{eqnarray*}

\begin{eqnarray*}High:\displaystyle \frac{\hat{\gamma }}{\widehat{{k}_{2a}}}> M* ,\end{eqnarray*}

where $\hat{\gamma }$ and $\widehat{{k}_{2a}}$ are the estimated values for $\gamma $ and ${k}_{2a},$ respectively. $\tfrac{\hat{\gamma }}{\widehat{{k}_{2a}}}$ is the estimated magnitude of DA release normalized to the estimated effective tracer efflux. It yields a unitless measure of peak release that can be easily compared across subjects (Normandin and Morris 2008, Normandin *et al* 2012). $M* $ is the critical scalar threshold separating low and high events.

We define characterization sensitivity (*CS*) as how precisely ${t}_{p}^{* }$ separates early and late events (*CS*_tp*_) or ${M}^{* }$ separates low and high events (*CS*_M*_). *CS*_tp*_ and *CS*_M*_ are defined mathematically as:

\begin{eqnarray*}\begin{array}{l}C{S}_{t{p}^{* }}=\displaystyle \frac{correct\,early\,classifications}{true\,early\,classifcations}+\,\displaystyle \frac{correct\,late\,classifications}{true\,late\,classifcations}=\displaystyle \frac{correct\,early\,classifications}{true\,early\,classifcations}\\ \,+\,\left(1-\,\displaystyle \frac{incorrect\,early\,classifications}{true\,late\,classifcations}\right)=\left(\displaystyle \frac{\,\displaystyle {\sum }_{\widehat{{t}_{p}}\in j}\left(\widehat{{t}_{p}}< {t}_{p}^{* }\right)}{| j| }\right)-\left(\displaystyle \frac{\,\displaystyle {\sum }_{\widehat{{t}_{p}}\in j}\left(\widehat{{t}_{p}}< {t}_{p}^{* }\right)}{| k| }\right)+1\end{array}\end{eqnarray*}

\begin{eqnarray*}\begin{array}{l}C{S}_{M* }=\displaystyle \frac{correct\,low\,classifications}{true\,low\,classifcations}+\displaystyle \frac{correct\,high\,classifications}{true\,high\,classifcations}=\displaystyle \frac{correct\,low\,classifications}{true\,low\,classifcations}\\ \,+\,\left(1-\displaystyle \frac{incorrect\,low\,classifications}{true\,high\,classifcations}\right)=\left(\displaystyle \frac{\,\displaystyle {\sum }_{\hat{M}\in m}\left(\widehat{M}< M* \right)}{| m| }\right)-\left(\displaystyle \frac{\,\displaystyle {\sum }_{\hat{M}\in n}\left(\widehat{M}< M* \right)}{| n| }\right)+\,1,\end{array}\end{eqnarray*}

where, *j* is the set of true early classifications, *k* is the set of true late classifications, *n* is the set of true low classifications, and *m* is the set of true high classifications.

*CS*_tp*_ values in the range [45 min, 55 min] were evaluated after classifying one-thousand $\widehat{{t}_{p}}$ values for each combination of simulated start-times and rise-times ($\widehat{{t}_{p}}-\widehat{{t}_{d}},$ i.e. the time between start-time and peak-time). *CS*_M*_ values in the range [0.6, 1.2] were evaluated after classifying one-thousand $\tfrac{\hat{\gamma }}{\widehat{{k}_{2a}}}$ ratios for each combination of simulated amplitude and rise-time.

### 2.5. Weighted k-nearest neighbor algorithm to reclassify voxels

A weighted k-nearest neighbor (wkNN) algorithm was developed to reclassify a voxel, based on the original classifications of its face-connected, edge-connected, and vertex-connected voxel neighbors. Voxels in each neighborhood, including the current voxel being reclassified, are weighted separately. Weights of the voxels in the neighborhoods were optimized for *DS*_*DA*_ using the Quasi-Newton algorithm (Shanno 1970). That is, the Quasi-Newton algorithm was applied to minimize the difference between the true positive signal randomly simulated in 10 sets of NS phantom data (construction of 4D phantom is described in 2.6), and the detected signal after estimation. Optimized weights for the current voxel, its faces, its edges, and its vertices were [1.00, 1.60, 1.14, 0.68], respectively. Classification is determined by the weighted vote of all neighboring voxels. The certainty of the classification can be assessed by the weighted vote for the final classification.

wkNN can be applied after voxel-level detection to improve image-level *DS*_*DA*_. It can also be used *after* voxel-level characterization of timing and amplitude to refine image-level characterization accuracy (*CA*). We define *CA* as:

\begin{eqnarray*}C{A}_{c}=\displaystyle \frac{correct\,classifcations}{total\,classifcations}=\displaystyle \frac{T{P}_{c}+T{N}_{c}}{T{P}_{c}+T{N}_{c}+F{P}_{c}+F{N}_{c}}\times 100 \% ,\end{eqnarray*}

where, *c* is a particular classification (e.g. early, late, high, or low), *TP* is the total number of true positive classifications, *TN* is the total number of true negative classifications, *FP* is the total number of false positive classifications, and *FN* is the total number of false negative classifications.

### 2.6. Detection and characterization of responses in 4D dynamic PET data

(1) Assignment of DA responses to a 3D parametric limbic phantom

**Figure 1.**
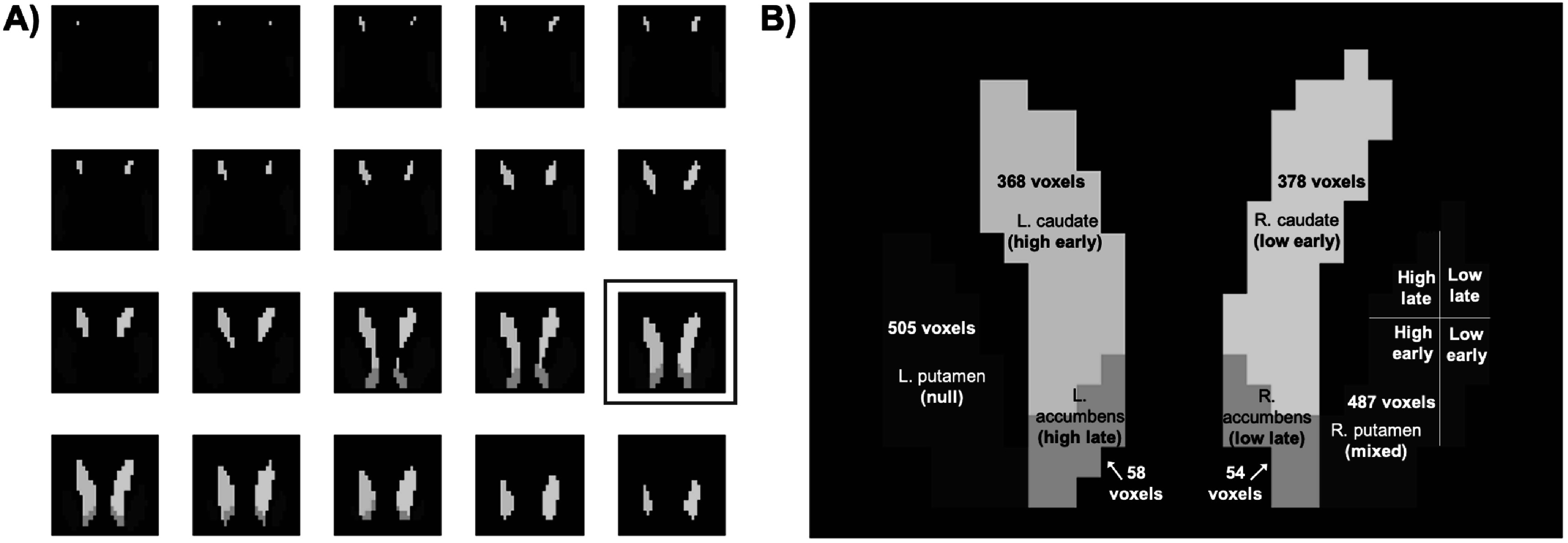
Anatomy of 3D limbic phantom. (A) All 20 coronal slices. (B) Selected slice (#15) labeled with the ground-truth classification(s) of the DA signals in each region; (i). L. putamen (505 total voxels), (ii). L. accumbens (58 total voxels), (iii). L. caudate (368 total voxels), (iv). R. caudate (378 total voxels), (v). R. accumbens (54 total voxels), (vi). R. putamen (487 total voxels).

**Figure 2.**
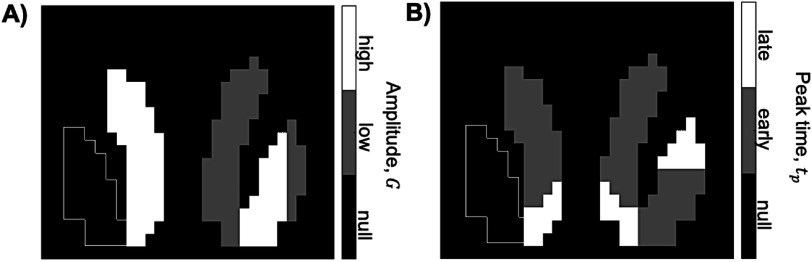
Ground-truth classification of (A) amplitude and (B) peak-time, in slice #15 of phantom.

The TPM neuromorphometrics atlas (found in SPM12: https://fil.ion.ucl.ac.uk/spm/toolbox/TPM/) was used to define the bilateral accumbens, bilateral putamen, and bilateral caudate. The regions were downsampled to roughly 2.5 mm × 2.5 mm × 2.5 mm voxel resolution, resulting in a phantom of 1850 total voxels (the bounding box of the image matrix was 29 × 20 × 17 voxels). Each voxel was assigned either a null response, or a response classified as high or low, and as early or late. Figure 1 shows the anatomy of the phantom and the ground-truth classification of the DA signal assigned to each voxel. Figure 2 shows the selected slice (shown in figure 1(B)) of the parametric images defining the ground-truth classification of amplitude (figure 2(A)) and peak-time (figure 2(B)).

The PET signals assigned to each voxel in a given class were not identical. Population variance was incorporated into the DA parameters by selecting parameters from probability distributions. Mean values for *G*_%_ and *t*_*p*_ were chosen based on the *DS*_*DA*_ determined in the first pilot study (see section 2.3). The ‘high’, ‘low’, ‘early’, and ‘late’ parameters were chosen such that the specified signals were marginally detectable in the HRRT, but reliably detectable in the NS.

Probability distributions and boundary conditions for all parameters in each class of DA responses were as follows:

1. **‘high’:** $G\,\sim \,N(400,\,50)$ [Normal distribution with (*μ*, *σ*)]
2. **‘low’:** $G\sim \,N(200,\,20)$
3. **‘early’:** ${t}_{p}\sim N$ (40 min, 3 min); $35\,\min \,< \,{t}_{p}< 75\,\min $
4. **‘late’:** ${t}_{p}\sim N$ (55 min, 10 min); $35\,\min \,< \,{t}_{p}< 75\,\min $
5. All classes: $\alpha \sim N(0.7,0.1)$
6. All classes: ${t}_{d}\sim \left[{t}_{p}-N\left(5,\,10\right)\right];35< {t}_{d}< {t}_{p}.$

**Figure 3.**
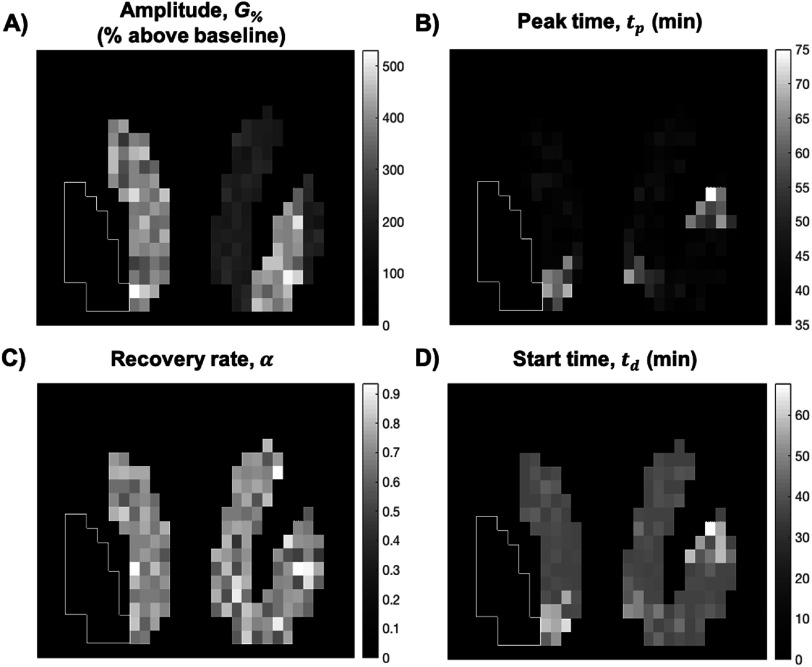
Simulated DA release parameters in phantom (shown for slice #15). (A) simulated amplitude, (B) simulated *t*_*p*_, (C) simulated *α*, (D) simulated *t*_*p*_. White outline delineates left putamen, which was populated entirely with null data. All other voxels within the bounding box contained no PET signal.

Figure 3 shows all simulated DA response parameters (needed for equation (2)) in slice 15 of the phantom. No population variance was incorporated into the tracer parameters, $\{{R}_{1},{k}_{2},{k}_{2a}\},$ in the simulation model.

(1) Simulation and analysis of phantom TACs
  Each voxel in the phantom (not including the bounding box) was assigned a TAC containing the DA response defined above. TACs were simulated using the methods described in section 2.1. Noise was generated to represent either the HRRT or the NS. Different levels of noise were explored for the NS. TACs with noise levels reflecting a 15-fold, 8-fold, and 6-fold decrease in measurement variance (relative to the HRRT) were simulated in separate phantoms. Each frame of dynamic phantom data was smoothed spatially with a 3D Gaussian filter (*σ* = 0.2 voxels) to introduce spatial correlation between voxels. *σ* was optimized for maximal *DS*_*DA*_in the phantom. All TACs within the striatal mask were fitted with MRTM and lp-ntPET using the same basis function library described in 2.2.
(2) Part 1: Detection of significant voxels
  A two-step process was implemented for *identifying* significant voxels. Step 1: ${\mathrm{\Delta }}BIC$ was calculated for each pair of fits (by lp-ntPET and MRTM) at each voxel. Voxels for which ${\mathrm{\Delta }}BIC< 0$ were classified as ‘significant’. Step 2: wkNN reclassification (to null or positive) was applied to all voxels to further eliminate false positives and increase *DS*_*DA*_. For comparison with wkNN, step 2 was eliminated and instead, a cluster-size threshold (CST) of 10 voxels was applied to voxels with ${\mathrm{\Delta }}BIC< 0.$ CST is the traditional method applied after voxel-level significance testing to eliminate false positives (Kim *et al* 2014).
(3) Part 2: Characterization of significant voxels.

A two-step process was also implemented for *characterizing* significant voxels. Step 1: equations (9) and (10) were applied to significant voxels to generate parametric images of the classifications for peak-time and amplitude. Step 2: wkNN reclassification was applied to the parametric images to reduce local heterogeneity and increase *CA*. The weights for wkNN were kept the same for Parts 1 and 2. The classification certainty was calculated for each significant voxel in the peak-time and amplitude images. The final classification statistics (*CA*, positive predictive value (PPV), and negative predictive value (NPV)) were calculated to assess the overall performance of the algorithm.

**Figure 4.**
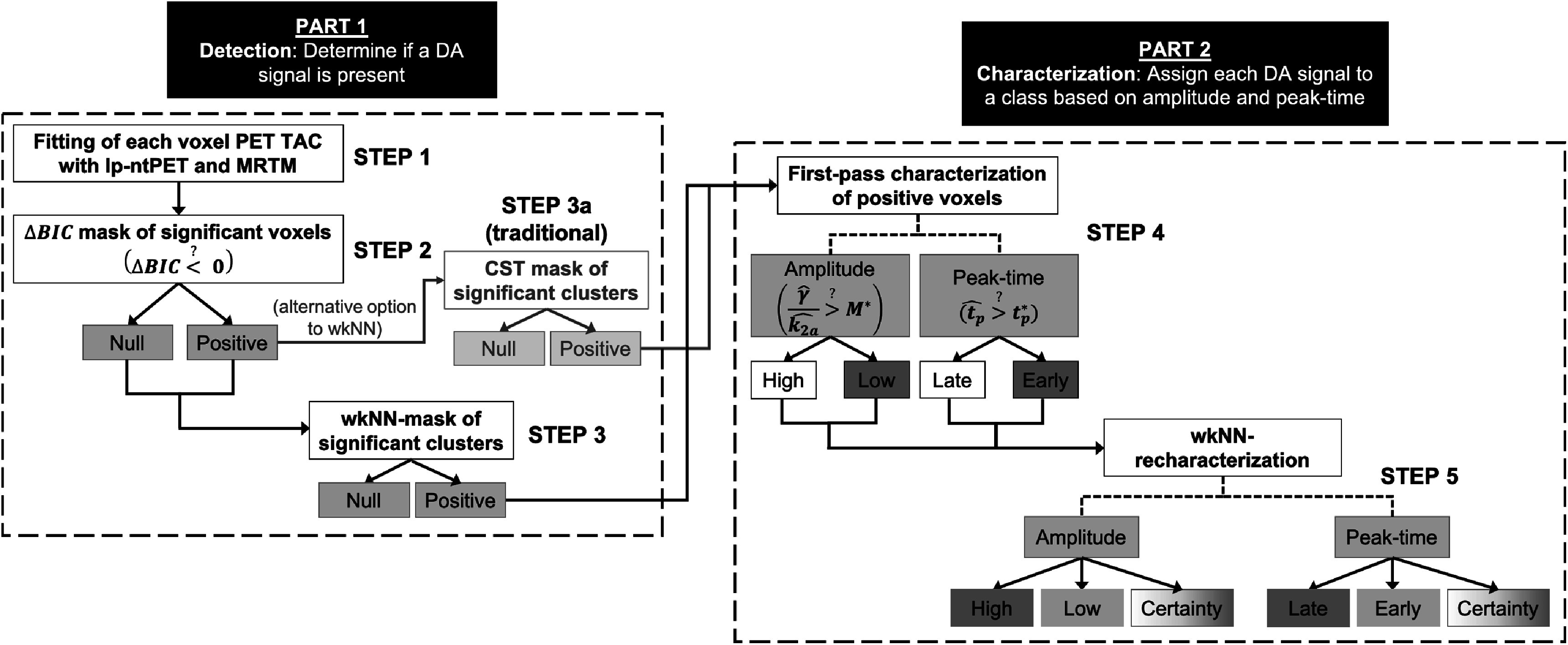
Summary of procedures used to detect and characterize DA signal in the simulated phantom data. The algorithm consists of two main parts: (1) detection and (2) characterization. Each part contains two main steps: (1) an initial classification using a thresholding method (*ΔBIC* in Part 1; M* and *t*_*p*_ * in Part 2) and (2) wkNN reclassification. CST may be applied in lieu of wkNN during the detection procedure. For comparison, both CST and wkNN were applied to the phantom data.

Figure 4 summarizes the methods applied in the detection and characterization of voxels containing a significant DA signal.

## 3. Results

### 3.1. Pilot study 1: *DS*_*DA*_ of future NS scanner to transient DA events

**Figure 5.**
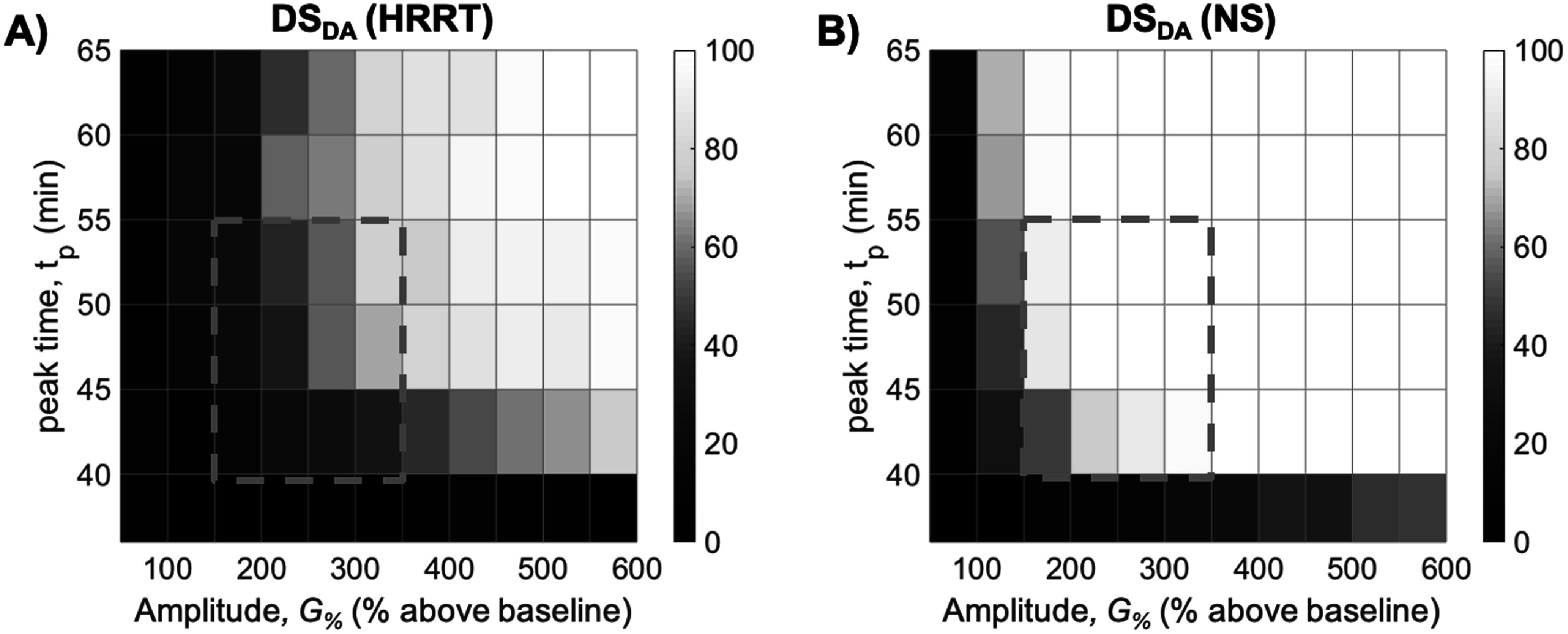
*DS*_*DA*_ of 1000 simulated responses for each combination of DA release amplitude and peak-time in (A) HRRT and (B) NS. Stimulus and response start-time is 35 min. *DS*_*DA*_ increases with *G*_%_ and *t*_*p*_ for both scanners. NS demonstrates increased range of signals for which *DS*_*DA*_ > 80%. Gray dashed outline delineates range of signals marginally detectable in HRRT, but reliably detectable in NS.

*DS*_*DA*_ of responses over a range of peak-times and amplitudes is shown in figure 5. *DS*_*DA*_ increased with higher response amplitude and peak-time. Responses with *t*_*p*_ < 40 min were detected with poor sensitivity (*DS*_*DA*_ < 50% for *G*_%_ > 600% at *t*_*p*_ < 40) in both scanners. In the simulated NS data, responses with *t*_*p*_ > 40 min were detected with *DS*_*DA*_ > 80% for *G*_%_ > 200%. The range of amplitudes and timing for which *DS*_*DA*_ = 100% (white area of figure 5) was much larger for the NS than the HRRT. The gray dashed box outlines a range of signals (*G*_%_ = [200%, 400%] above baseline, *t*_*p*_ = [40 min, 55 min]) that are marginally detectable in the HRRT, but reliably detectable in the NS.

### 3.2. Pilot study 2: performance of classification thresholds

**Figure 6.**
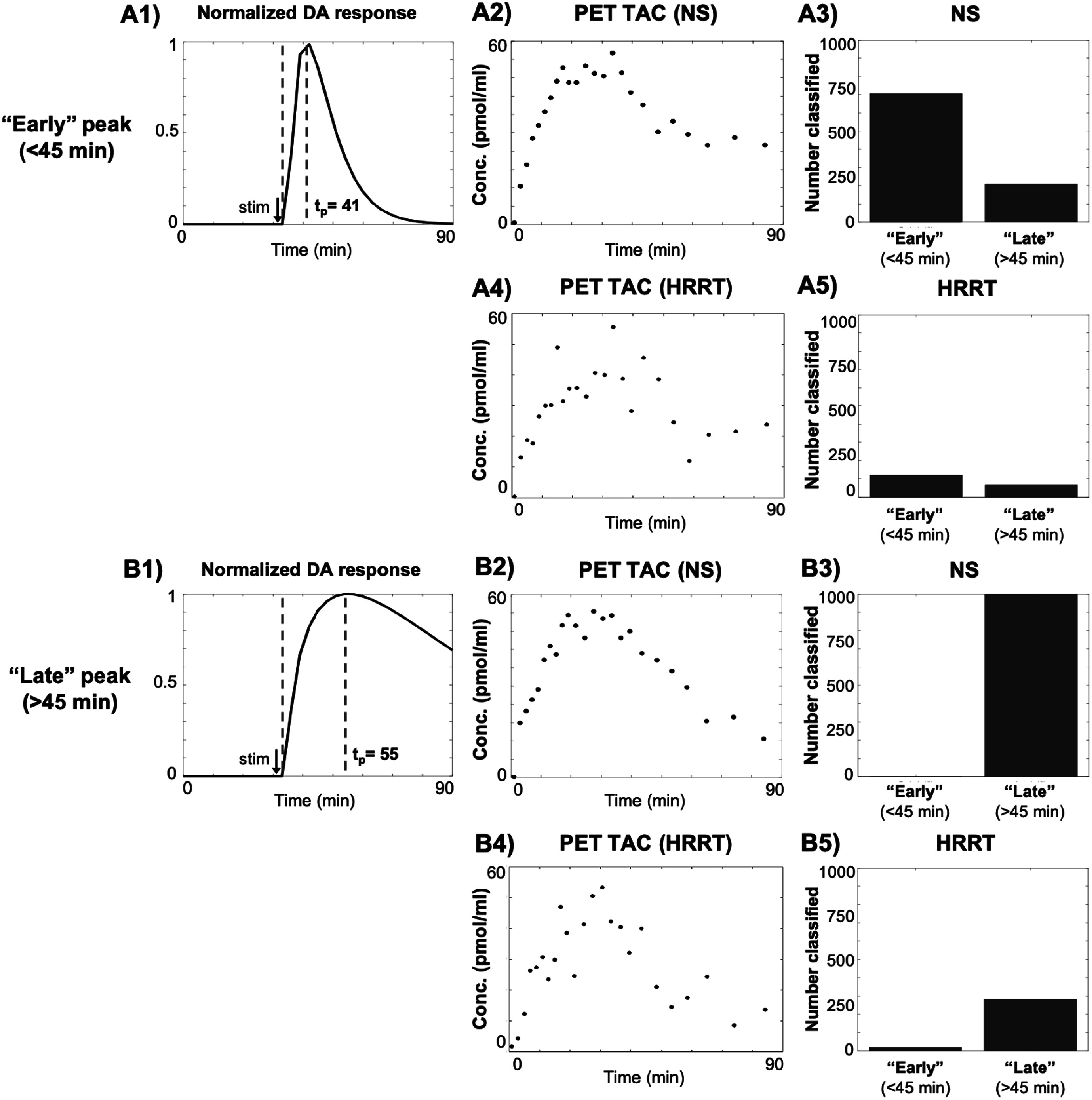
Example of classification of (A) early-peaking (${t}_{p}$ = 41 min) and (B) late-peaking (${t}_{p}$ = 55 min) DA responses. Responses start at 35 min and have an amplitude of 250% above baseline. Panels A1 and B1: normalized simulated DA response. Red dashed lines indicate simulated values for ${t}_{p}$ and ${t}_{d},$ respectively. Panels A2, A4, B2, and B4: simulated voxel-level time-concentration (specific activity = 634 MBq nmol^−1^) PET curves for NS and HRRT. Panels A3, A5, B3, and B5: bar graphs for ‘early’ and ‘late’ classifications using ${t}_{p}^{* }$ = 45 min. Total detected responses (out of 1000 total) is the sum of the two bars.

Figure 6 shows the distribution of voxels classified as ‘early’- and ‘late’-peaking for two examples of simulated responses with *t*_*d*_ = 35 min. One example (figure 6(A)) peaked at 41 min (6 min post-stimulus), and the other (figure 6(B)) peaked at 55 min (15 min post-stimulus). Both responses had *G*_%_ = 250%. For classification, ${t}_{p}^{* }$ was arbitrarily chosen to be 45 min. Note that here, *DS*_*DA*_ = $\tfrac{total\,early\,classifications+total\,late\,classifcations}{1000\,simulated\,signals}\times 100 \% .$ Of the 1000 simulated TACs for the early-peaking response, *DS*_*DA*_ for the NS was 89%, compared with 21% for the HRRT. For the late-peaking response, *DS*_*DA*_ for the NS was 100%, compared with 38% for the HRRT.

Of the early-peaking responses detected by the NS scanner, 72% were correctly classified as ‘early’. Of the late-peaking responses detected by the NS scanner, 100% were correctly classified as ‘late’.

**Figure 7.**
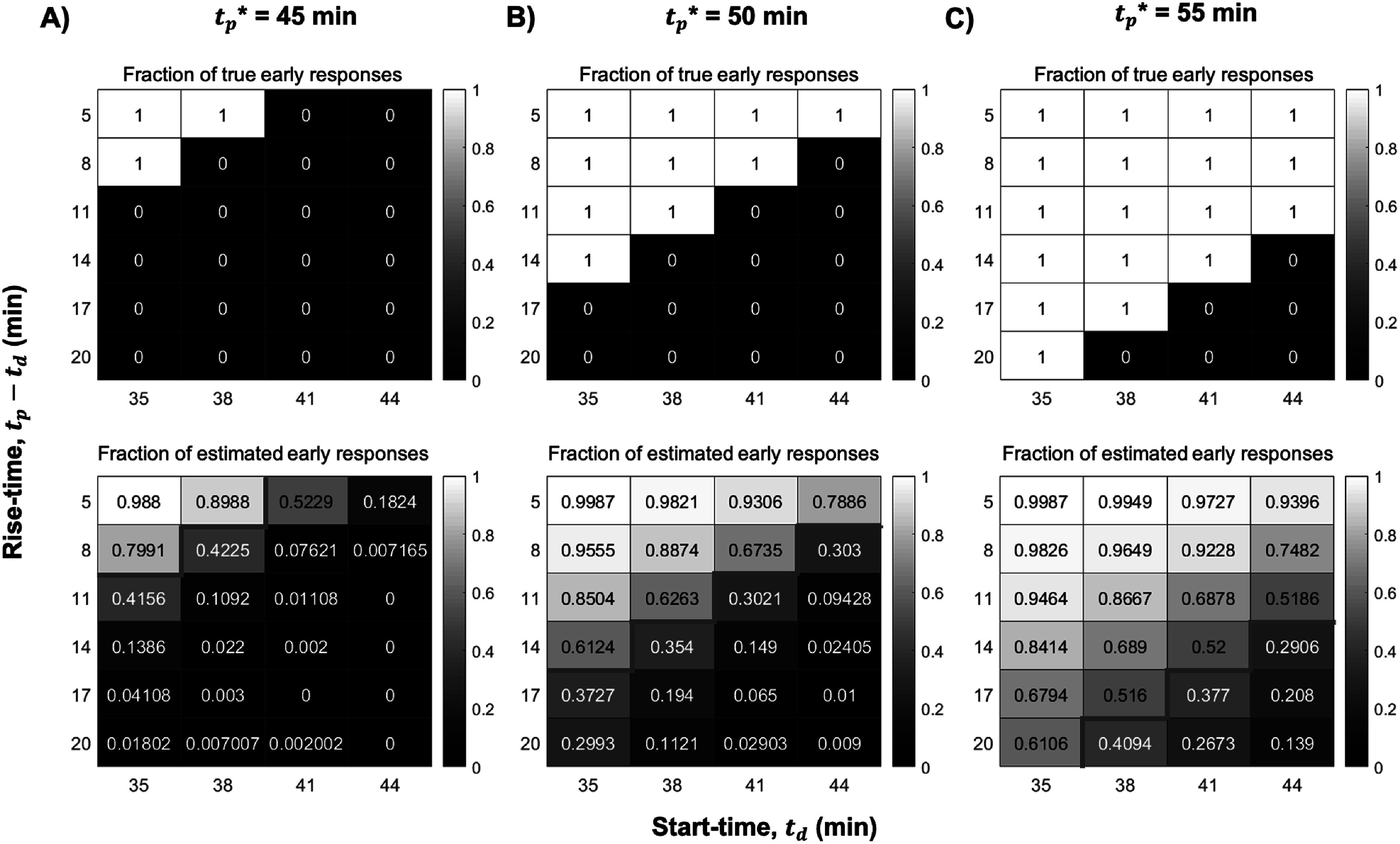
Fraction of ‘early’ classifications using different *t*_p_* thresholds in NS data. *G*% = 200% for all heatmaps shown. (A) *t*_p_* = 45 min, (B) *t*_p_* = 50 min, (C) *t*_p_* = 55 min. Ordinate of heatmaps is the ‘rise-time’ of the response, i.e. *t*_*p*_ - *t*_*d*_. Top row shows the true fraction of simulated responses peaking earlier than *t*_p_*. Bottom row shows the fraction of responses with *estimated* peak-times before *t*_p_* (classified as ‘early’). All responses not classified as ‘early’ are classified as ‘late’. *CS*_tp*_ is indicated by distinctness of separation between early and late events (see the *red border*). The red border indicates the ground-truth threshold, ${t}_{p}^{* },$ that separates simulated ‘early’ and ‘late’-peaking signals. The optimal *CS*_tp*_ produces the sharpest transition between high- and low-intensity gray-levels at the red border.

Figure 7 shows the fraction of simulated DA responses characterized as ‘early’, based on three possible ${t}_{p}^{* }$ thresholds in the NS data. The ordinate of the heatmaps indicates the rise-time ($\widehat{{t}_{p}}-\widehat{{t}_{d}},$ i.e. the time between start-time and peak-time) of the simulated response. The abscissa indicates the start-time. All events not classified as ‘early’ are, by definition, classified as ‘late’. *CS*_tp_* can be assessed visually in figure 7. The threshold, ${t}_{p}^{* },$ with the highest *CS*_tp*_ provides the sharpest separation of early- and late-peaking events, and most closely reproduces the true classification of early versus late events. Figure 7 shows that the later the ${t}_{p}^{* },$ the more diffuse is the border between estimated early-peaking and late-peaking responses, and the lower the *CS*_tp*_. Visually, this is indicated by the less distinct border between high- and low-intensity gray-level along the diagonal of the heatmaps in figure 7.

${t}_{p}^{* }$ = 45 min was chosen to separate early-peaking from late-peaking events. ${t}_{p}^{* }$ = 45 min allows an adequate 10 min window for events to peak ‘early’ at various times after a stimulus at 35 min, while maintaining the highest *CS*_tp*_ given this peak-time window.

Figure S2 shows the fraction of simulated DA responses characterized as ‘low’ based on three possible ${M}^{* }$ thresholds. The threshold, M*, with the highest *CS* _M*_ provides the sharpest separation between ‘low’ and ‘high’ signals (simulated with *G*_%_ = 200% and *G*_%_ = 400%, respectively). Characterization of estimated amplitude was most dependent on simulated amplitude, rather than rise-time or start-time (not shown), as indicated by the evident gradient in the direction of the abscissa, but relatively uniform intensity along the ordinate.

*CS*_M*_ was highest for ${M}^{* }$ = 0.8, and was chosen to separate low-amplitude from high-amplitude events.

### 3.3. Detection and classification of responses in 4D dynamic PET phantom

**Figure 8.**
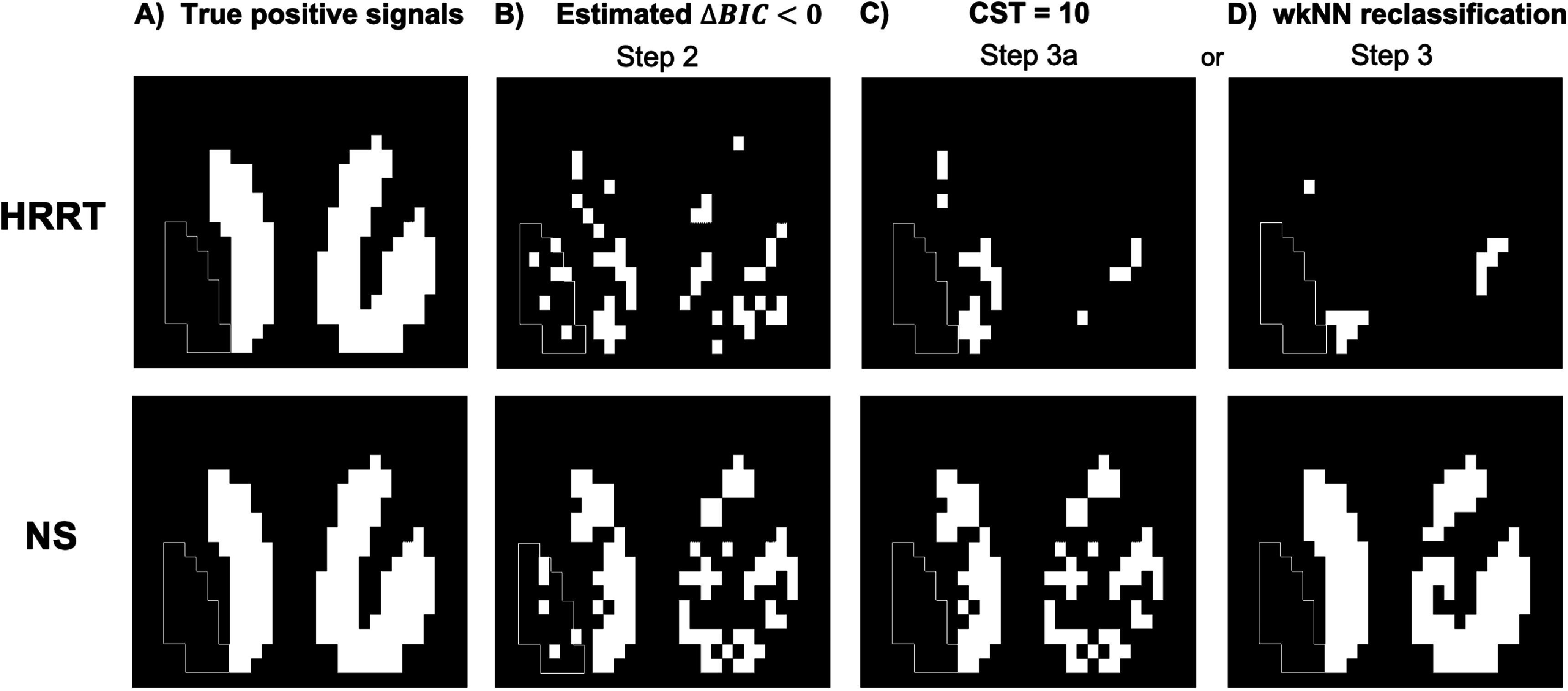
Voxels with significant responses after applying step 2, and step 3 *or* 3a of algorithm (figure 4) to HRRT phantom data (top) and NS phantom data (bottom). Shown in slice #15: (A) true positive voxels, (B) voxels with ${\mathrm{\Delta }}BIC< 0,$ (C) remaining voxels after applying CST = 10 to column B, and (D) reclassified voxels after applying wkNN to column B. White outline delineates left putamen, which contained only null data.

Table 1 summarizes the detection statistics after ${\mathrm{\Delta }}BIC$-masking (figure 4, Step 2), and then after *either* CST *or* wkNN-masking (figure 4, Steps 3a and 3). Figure 8 shows binary images of true-positive voxels and significant detected voxels after each step. All significant voxels found in the left putamen are false positives because no DA responses were simulated in the TACs in that region. The detection specificity of ${\mathrm{\Delta }}BIC< 0$ was 92% in the NS scanner and 89% in the HRRT. The detection sensitivity (*DS*_*DA*_) was 72% in the NS scanner and 31% in the HRRT. Both wkNN reclassification and CST increased detection specificity to 100% in both the NS scanner and HRRT, which translates to a false positive rate of 0%. wkNN reclassification increased *DS*_*DA*_ from 72% to 92% in the NS scanner; however wkNN actually decreased *DS*_*DA*_ from 31% to 10% in the HRRT.

**Table 1.** Detection statistics in HRRT and NS phantom.

Detection performance	${\mathrm{\Delta }}BIC< 0$	CST = 10	wkNN reclassification
Detection sensitivity (*DS*__*DA*__), HRRT	31%	13%	10%
Detection sensitivity (*DS*__*DA*__), NS	72%	71%	92%
Detection specificity, HRRT	89%	100%	100%
Detection specificity, NS	92%	100%	100%

**Figure 9.**
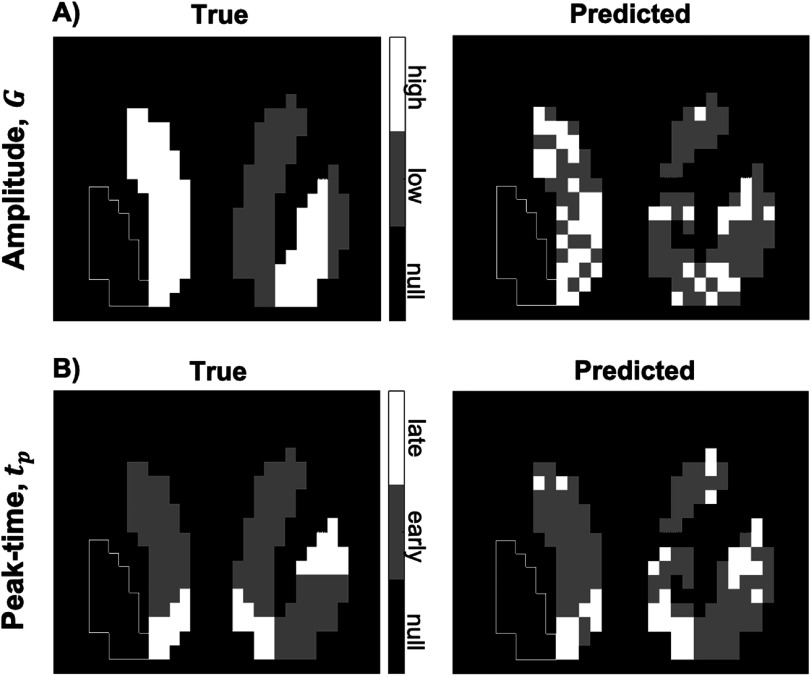
True classifications (left) and predicted classifications (right) using ${t}_{p}^{* }=45\,\min $ and $M* \,=\,0.8$ (figure 4, Step 4). In slice #15 of the phantom: (A) amplitude, (B) peak-time.

Figure 9 shows the characterization of significant voxels for peak-time and amplitude, using equations (7) and (8) (figure 4, Step 4). Misclassification of high- versus low-amplitude voxels (figure 9(A), right) is more apparent than early- versus late-peaking voxels (figure 9(B), right), suggesting (*CA*_*high*_, *CA*_*low*_) < (*CA*_*early*_, *CA*_*late*_). Specifically, high-amplitude voxels were often misclassified as low-amplitude.

**Figure 10.**
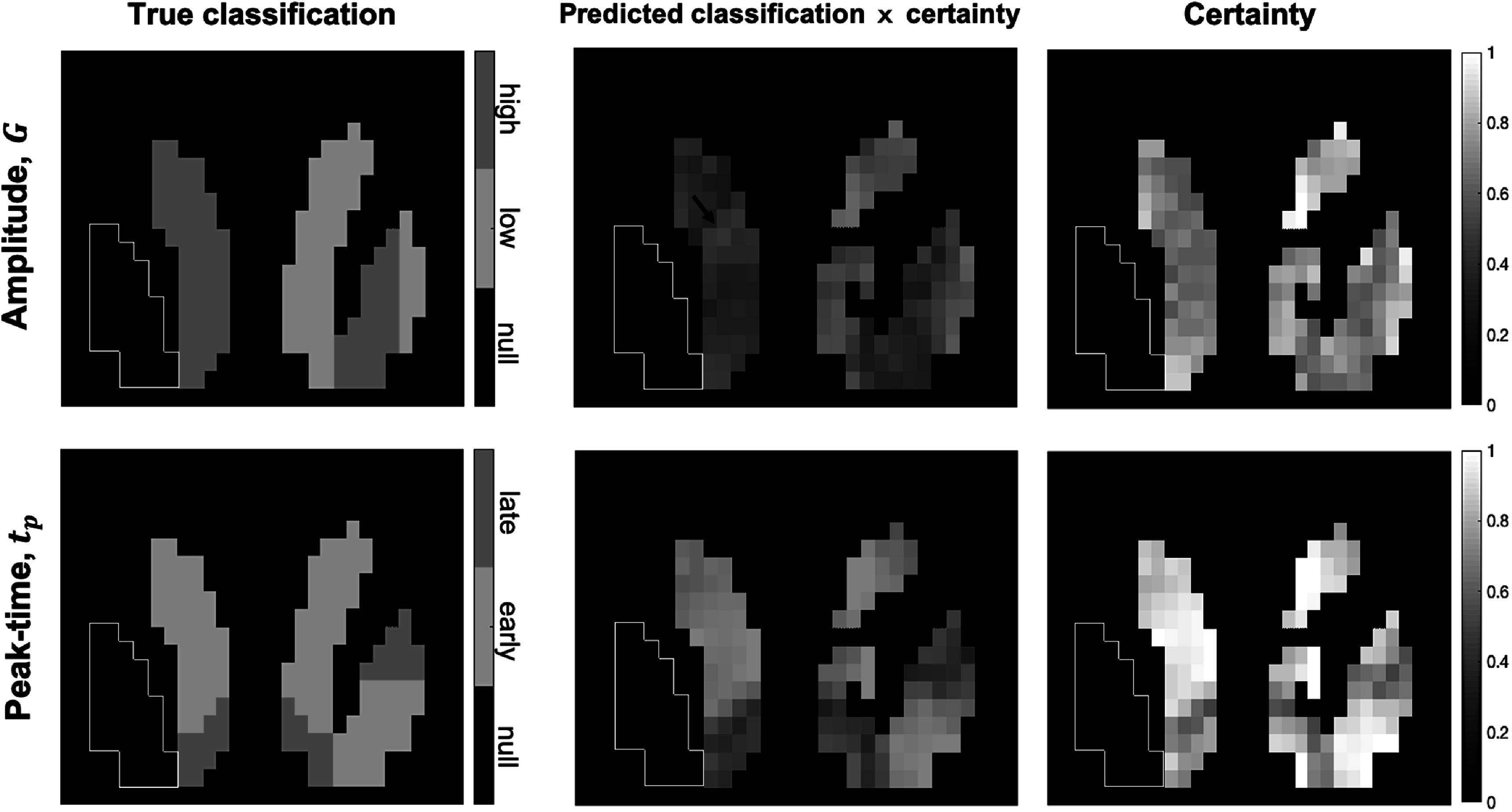
True classification (left), final predicted classification (middle), and certainty of predicted classification (right) of amplitude (top) and peak-time (bottom) after wkNN reclassification (figure 4, Step 5) in slice #15 of phantom. Color intensity of significant voxels indicates certainty of classification. Arrow in predicted amplitude image indicates location of large cluster where high-amplitude signals in the L. putamen and L. accumbens were misclassified as low-amplitude.

Figure 10 shows the final characterizations and certainty of characterizations of voxels after wkNN reclassification of the NS phantom (figure 4, Step 5). Low certainty scores indicate less certainty at the borders between different predicted classifications, and where the predicted classifications were incorrect (e.g. the L. putamen and L. accumbens in the amplitude image; see arrow in figure 10). Overall, peak-time is classified with greater certainty than amplitude.

Table 2 summarizes characterization statistics. Classification of detected signals with high-amplitude and early-peak-time had a PPV >97%, meaning that of the signals classified as high-amplitude and/or early-peaking, over 97% were correct. This result indicates that <3% of true low-amplitude and true late-peak-time signals were misclassified as high-amplitude or early-peak-time, respectively. Late-peaking signals had a NPV of 99%, meaning that of the signals *not* classified as late-peaking, only 1% were truly late-peaking. Early-peaking and high-amplitude signals had higher *CA* than late-peaking and low-amplitude signals, respectively.

**Table 2.** Characterization statistics in NS phantom.

Classification performance	Amplitude high	Amplitude low	Peak-time early	Peak-time late
Positive predictive value ($PPV=\tfrac{TP}{TP+FP}$)	97.24%	80.66%	98.22%	95.17%
Negative predictive value $(NPV=\tfrac{TN}{TN+FN})$	88.47%	89.45%	86.32%	98.95%
False discovery rate ($FDR\,=\,1\mbox{--}\,PPV=\tfrac{FP}{TP+FP}$)	2.76%	19.34%	1.78%	4.83%
False omission rate ($1-NPV=\tfrac{FN}{TN+FN}$)	11.53%	10.55%	13.68%	1.05%

#### 3.3.1. Robustness of characterization accuracy to noise assumptions

*CA* degraded only slightly with decreased improvement in *DS*_*counts*_. For a 6-fold increase in *DS*_*counts*_ relative to the HRRT (rather than the 10-fold relative increase expected for the NS), *CA*_*late*_ and *CA*_*low*_ were 98% and 73% (versus 98% and 86% in NS), respectively. *CA* of all classifications in phantoms with 15-fold, 10-fold (NS) 8-fold, and 6-fold increase in *DS*_*counts*_ (relative to the HRRT) can be found in figure S5 and table SI in the supplemental document.

## 4. Discussion

### 4.1. Estimation of timing information in PET: From ROI to voxel

lp-ntPET is a linearized version of ntPET, ideal for use at the voxel level. However, at the voxel level, SNR is reduced. In PET, SNR is dictated by the sensitivity of the scanner to radioactive events. The anticipated NS scanner is expected to have a 10-fold improvement in *DS*_*counts*_ over the current state-of-the-art, with a commensurate improvement in SNR. Our simulations were designed with these improvements in mind. Our classification of simulated data supports the hypothesis that the future NS scanner will expand the range of detectable DA signals appreciably, and make possible the characterization of signals based on DA amplitude and peak-time at the voxel-level.

The effect of transient endogenous competition on the estimation of receptor availability from PET data has been well-modeled for some time, beginning possibly with work by Logan *et al* (1991). An early model posed by Morris *et al* (1995) and Fisher *et al* (1995) incorporated time-varying DA into a compartmental model for RAC uptake, and proposed a metric to assess the degree of DA activation. Exploration of a similar model by Endres and Carson (1998) suggested that the calculated volume of distribution of the tracer is related to the integral of an endogenous NT pulse. This concept was expanded by Morris and Yoder (2007) to further quantify the sensitivity of PET to assay fluctuations of endogenous NT (a.k.a., ‘PET displacement sensitivity’). The PET displacement sensitivity was found to consist of multiple factors, including the temporal dynamics of the competitor (i.e. the endogenous NT). Sullivan *et al* (2013) subsequently demonstrated that traditional time-invariant PET models, whose outcome is steady-state receptor availability (*BP*_*ND*_), are biased by changes in endogenous NT. The demonstrated deficiences in traditional time-invariant kinetic models motivated the development of the ntPET models. These models were configured to include parameters for both the steady-state kinetics of the tracer and the temporal characteristics of a transient NT signal (Morris *et al* 2005, Constantinescu *et al* 2008, Morris *et al* 2008, Normandin and Morris 2006, 2008, Normandin *et al* 2012, Kim *et al* 2014, Wang *et al* 2017).

### 4.2. Characterization of detected DA events

We recognize that there is an arbitrariness to the designation of peak-time as the sole characteristic of timing. However, our first goal was to simplify the classification problem to its bare essentials. In the future, one might consider some combination of [${t}_{d},\,{t}_{p},\,\alpha $] as classifiers. *t*_*d*_ represents the initiation of a biological response and *t*_*p*_ is the time of maximal response. Either or both might be important. One must recognize that any timing classifier becomes less precise later in the scan because of the progressively decreasing SNR in PET data. This loss of precision was reflected in the *CS*_tp*_, as shown in figure 7.

The magnitude classifier, ${M}^{* },$ performed less reliably than the timing classifier, ${t}_{p}^{* }.$ This is indicated by a higher percentage of misclassified ‘low’ versus ‘high’ responses compared to ‘early’ versus ‘late’ responses even at optimal thresholds (see figure 10 and table 2). In this work, we chose ${M}^{* }$ = 0.8 given *a priori* knowledge that there was one simulated distribution of ‘low’ amplitudes centered at 200% above baseline and another simulated distribution of ‘high’ amplitudes centered at 400% above baseline in the phantom. In practice, however, determining an optimal ${M}^{* }$ *a priori* may not be possible because the range of amplitudes may not be known and the true distribution of $\displaystyle \frac{\gamma }{{k}_{2a}}$ may not be bimodal. Thus, it may be better to examine the distribution of $\tfrac{\hat{\gamma }}{\widehat{{k}_{2a}}}$ and define $M* $ *a posteriori*. During this process, it should also be considered that higher-amplitude events have a greater chance of passing significance testing and thus being detected. Thus, the distribution of significant $\tfrac{\hat{\gamma }}{\widehat{{k}_{2a}}}$ may be more skewed towards higher values than the true distribution of $\tfrac{\gamma }{{k}_{2a}}.$

### 4.3. Performance of the wkNN algorithm

**Figure 11.**
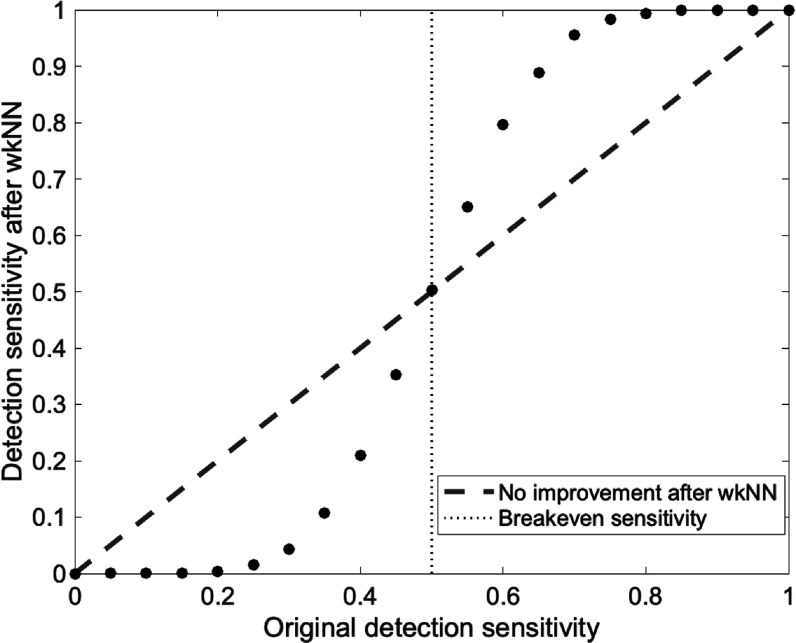
*DS*_*DA*_ after wkNN reclassification versus *DS*_*DA*_ before wkNN reclassification. Each data point represents the average of 10 phantoms (1850 voxels each) with randomly distributed detected voxels at the indicated detection fraction. Red slope indicates no change in *DS*_*DA*_ after wkNN. If the original *DS*_*DA*_ is under 50%, wkNN degrades *DS*_*DA*_. If the original *DS*_*DA*_ is above 50%, wkNN improves *DS*_*DA*_.

The success of wkNN in refining the classification of simulated NS scanner data is dependent on the ultra-high *DS*_*counts*_ of the scanner, which makes possible a *DS*_*DA*_ over 50% prior to reclassification. Figure 11 shows the relationship between *DS*_*DA*_ before wkNN reclassification and after wkNN reclassification. High *DS*_*DA*_ is a *sine qua non* for success of the algorithm because sufficient information must be contained in the 27-voxel-neighborhood of any voxel of interest to hold a meaningful vote. In the HRRT, most voxels initially classified as positive will be (incorrectly) reclassified as null because most of the voxels’ 27-neighborhood members will be null. The certainty of classification is an interim step within wkNN that may offer additional value beyond classification alone. The certainty scores reflect the degree of consensus of the neighborhood in characterizing a positive voxel—the interpretation being that higher consensus of the neighborhood connotes higher confidence in the final (re)classification.

The wkNN algorithm appears to have been successful in both detection and characterization of DA signals (see figures 8(D), 10 and S3 and S4). wkNN is superior to traditional CST in simulated NS data because CST determines if a positive voxel is retained based on the number of voxels connected to it, while wkNN uses a weighted vote of a positive or null voxel’s entire 27-neighborhood. Consequently, CST can only eliminate false positive voxels, while wkNN can both eliminate false positive voxels and restore false negative voxels.

wkNN is distinct from spatial smoothing. The input of the wkNN algorithm is a binary map, for which smoothing is not appropriate. wkNN is preferred because it can recover false negative voxels (figure 8(D)), and returns a probability (the result of the vote) for a given classification.

### 4.4. Assumptions and limitations

#### 4.4.1. Measurement variance

All the predictions we present for the performance of the NS assume a 10-fold increase in *DS*_*counts*_ compared to the HRRT. We examined the impact of this assumption on our findings. The proposed NS hardware will incorporate the long-AFOV systems developed by the EXPLORER consortium, depth-of-interaction-based reconstruction, and greatly-improved time-of-flight resolution from an application-specific integrated circuit (Poon *et al* 2012, Cherry *et al* 2017, Berg *et al* 2018, Cherry *et al* 2018). Simulations by the consortium (beyond the scope of this work) were used to justify the order-of-magnitude increase in sensitivity assumed for this work. There are various ways to make use of the 10-fold increase in sensitivity. For our purposes, we chose to apply all of the improvement to reduced measurement variance (while maintaining the dose, frame duration, scan length, and reconstructed voxel size commonly used with the current technology).

Fortunately, the improvement in performance is fairly robust to the choice of noise level. We found that downgrading the sensitivity of the NS to 6-fold that of the HRRT degraded the accuracy of classifications only minimally. Figure S5 in the supplemental document illustrates the insensitivity to exact choice of SNR within a reasonable operating range. Thus, we believe that the results presented herein are valid for predicting the utility of any future noteworthy improvement in brain scanner sensitivity.

#### 4.4.2. DA signal parameters

The DA signals simulated in the work reflect a limited set of all physiologically possible signals. The timing and amplitude parameters were chosen to represent endogenous DA release in response to a moderate pharmacological stimulus. The intent was to emulate the stimulant-induced DA release profiles measured from microdialysis experiments (Carboni *et al* 1989, Laruelle *et al* 1997, Morris *et al* 2008). Similar timing and amplitude parameters have been used in other ntPET simulation studies (Morris *et al* 2005, Normandin and Morris 2006, 2008, Normandin *et al* 2012, Wang *et al* 2017, Angelis *et al* 2019, Hu *et al* 2019, Bevington *et al* 2020, Fuller *et al* 2020).

Detectability of the DA signal is affected by variables other than peak-time and amplitude. Detection sensitivity is driven, in part, by the area under the DA curve (AUDC), which depends on the total amount of DA release during the scan. Given our assumption that the start-time of the signal is bounded on the lower end by the stimulus-time, the early-peaking signals tend to have smaller AUDC. This causes the variability in peak-time to be greater than the variability in start-time. Greater variability in peak-time leads to an inevitable bias of larger AUDC for later peak-times, probably contributing to greater detectability of late-peaking signals.

#### 4.4.3. Noise model

For this work, Gaussian noise with variance reflecting Carbon-11 decay was added independently to each noiseless simulated TAC (Mazoyer *et al* 1986). Positive correlation between voxels was introduced via Gaussian smoothing. A more realistic simulation would involve forward-projecting an activity distribution into sinogram space using a scanner-specific forward model, adding Poisson variance directly to the sinogram, and back-projecting to produce noisy TACs (Angelis *et al* 2015, Angelis *et al* 2019). The latter approach would result in a more realistic pattern of noise correlation between voxels than we have created here. But absent a functioning NS scanner, we chose a simpler approach to test our hypothesis. A more appropriate noise model may be established once the NS has been built and its noise properties have been measured.

#### 4.4.4. wkNN weights

The wkNN weights were optimized for detection sensitivity (true positive rate) based on the binary detection mask returned at Step 2 (figure 4). Unfortunately, the detection masks for the HRRT were very sparse (figure 8(B)) and optimization failed. We recognize that applying weights optimized for the NS will bias results in favor of the NS.

#### 4.4.5. Motion correction

The authors are not addressing motion correction in the current work. However, motion correction in any future scanner must be addressed as is the case for any current scanner. Motion correction in any NS will need to be at least as successful as methods used in concert with the HRRT.

#### 4.4.6. Scanner design configuration

The authors have not explored the various possible design configurations of the scanner, which is tangential to the current work. Other groups have done extensive work to demonstrate that >10-fold improvement in sensitivity can be achieved through different designs (Catana 2019, Carson *et al* 2021). Sensitivity is defined as counts captured per counts emitted. If sensitivity goes up, SNR goes up—for a fixed injection, fixed time bins, fixed voxel size.

### 4.5. Potential applications of the NS

The anticipated NS scanner could be a powerful tool to study the kinetic heterogeneity of DA transients in the limbic system. Microdialysis and cyclic voltammetry studies have revealed the diverse temporal profiles of DA signals. This diversity of DA patterns may underlie function and dysfunction within the brain’s microenvironment (Coe *et al* 2005, Rollema *et al* 2007, Jedema *et al* 2014, Narendran *et al* 2014, Taylor *et al* 2015, Walters *et al* 2016). Unfortunately, microdialysis and cyclic voltammetry are invasive and rarely used in humans. However, it would be of great interest to search for evidence of kinetic heterogeneity in humans at the voxel level, *in vivo.* The NS might allow researchers to explore the presence of a macroscopic analog of kinetic variability of neurochemical signals that exists at the neuronal scale. We consider one translational and one basic research example, here.

#### 4.5.1. Drug development

Varenicline, a nicotinic acetylcholine receptor partial agonist, is commonly prescribed as a smoking cessation aid. Unfortunately, less than 30% of patients achieve long-term abstinence from smoking (Agboola *et al* 2015, Taylor *et al* 2017). Why is this the case? Microdialysis studies in rats have demonstrated that varenicline attenuates the amplitude and delays the peak-time of nicotine-induced DA release (Rollema *et al* 2007). The effect of varenicline on the spatiotemporal signature of DA release may be crucial to understanding the therapeutic mechanisms of the drug. The ability to use PET to characterize the peak-time and amplitude of smoking-induced DA release—and any medication-induced changes—could help elucidate the relationship between the neurochemistry and the efficacy of varenicline. Amplitude and peak-time images in humans could test the hypothesis that the drug curbs nicotine craving by altering local DA dynamics. A clinical trial of varenicline using amplitude and peak-time images as endpoints might predict response to treatment or inform the development of the next generation of therapies. Broadly, the NS scanner equipped with a classification algorithm, such as the one proposed in this work, might empower scientists to test hypotheses regarding how drugs affect NT dynamics in the living human brain.

#### 4.5.2. Neurochemical ‘connectivity’

Characterization of voxels based on their DA timing parameters allows for grouping of temporally coherent voxels, independent of their spatial contiguity. Voxels with similar time courses of DA release in response to a certain stimulus might be interpreted as neurochemically ‘connected’. The np-ntPET model (Constantinescu *et al* 2007) was previously used to identify striatal voxels connected by their synchronous DA responses to finger tapping (Morris *et al* 2010). The visualization of these voxels can be considered a novel type of PET image that is a foreruner of the images produced herein. Morris *et al* (Morris *et al* 2010) identified connected voxels by their peak-time. Here we applied a formal classification approach to a similar type of data.

Connectedness of voxels is a framework to aid in identifying brain areas with similar timing of DA *release*. However, it can also be a way to visualize DA *inhibition*. In a study by (Ko *et al* 2008), transcranial magnetic stimulation (TMS) was used to briefly inhibit dopaminergic function in the left dorsolateral prefrontal cortex (DLPFC). It was discovered that TMS inhibition in the DLPFC also inhibited task-based DA release in the bilateral striatum. The parallel disruption of DA transmission in both the DLPFC and the striatum suggests that these spatially distinct regions are in fact neurochemically connected. It is not surprising that DA function in these regions, which comprise the mesocorticalimbic circuit, are responding in unison. The algorithm proposed in this work could be used in conjunction with the NS scanner to explore how the mesocorticolimbic system activates certain DA ‘circuits’ in response to various stimuli.

## 5. Conclusion

The next-generation of brain PET scanners is imminent. With greatly-improved sensitivity to radioactive events, the NS will make it possible to probe NT transmission in ways that are not possible with the current state-of-the-art. We have demonstrated the success of a novel algorithm for classifying DA signals in simulated data. Coupled with newly-achieved ultra-high sensitivity, the algorithm will make it possible to classify DA peak-time and amplitude at the voxel-level resolution. Using this next generation of hardware and computational tools, scientists will be able to elucidate the unique spatiotemporal patterns of NT signals that likely underlie some psychiatric diseases and human behaviors. The rationale for new brain scanners is not solely to image dopamine kinetics. Increased sensitivity in dynamic PET will make possible many new observations, some of which cannot yet be contemplated.

## Appendix. List of key abbreviations

**Abbreviation Definition**
CA: Characterization accuracy
CS_M* _: Characterization sensitivity to high/low events
CS_tp***_: Characterization sensitivity to early/late events
DA: Dopamine
DS_counts_: Detection sensitivity to radioactive events
DS_DA_: Detection sensitivity to dopamine signals
G: Concentration of dopamine at peak of release
G_%_: Percentage above baseline of dopamine at peak of release
NT: Neurotransmitter
t_d_: Dopamine signal start time (relative to injection)
t_p_: Dopamine signal peak time (relative to injection)
